# Short-term associations between ambient air pollution and emergency department visits for Alzheimer’s disease and related dementias

**DOI:** 10.1097/EE9.0000000000000237

**Published:** 2022-12-22

**Authors:** Haisu Zhang, Liuhua Shi, Stefanie T. Ebelt, Rohan R. D’Souza, Joel D. Schwartz, Noah Scovronick, Howard H. Chang

**Affiliations:** a Gangarosa Department of Environmental Health, Rollins School of Public Health, Emory University, Atlanta, Georgia; b Department of Biostatistics and Bioinformatics, Rollins School of Public Health, Emory University, Atlanta, Georgia; c Department of Environmental Health, Harvard T.H. Chan School of Public Health, Boston, Massachusetts.

**Keywords:** Air pollution, Emergency department visits, Hospitalization, Alzheimer’s disease, Dementia, Health effect

## Abstract

Dementia is a seriously disabling illness with substantial economic and social burdens. Alzheimer’s disease and its related dementias (AD/ADRD) constitute about two-thirds of dementias. AD/ADRD patients have a high prevalence of comorbid conditions that are known to be exacerbated by exposure to ambient air pollution. Existing studies mostly focused on the long-term association between air pollution and AD/ADRD morbidity, while very few have investigated short-term associations. This study aims to estimate short-term associations between AD/ADRD emergency department (ED) visits and three common air pollutants: fine particulate matter (PM_2.5_), nitrogen dioxide (NO_2_), and warm-season ozone.

**Methods:**

For the period 2005 to 2015, we analyzed over 7.5 million AD/ADRD ED visits in five US states (California, Missouri, North Carolina, New Jersey, and New York) using a time-stratified case-crossover design with conditional logistic regression. Daily estimated PM_2.5_, NO_2,_ and warm-season ozone concentrations at 1 km spatial resolution were aggregated to the ZIP code level as exposure.

**Results:**

The most consistent positive association was found for NO_2_. Across five states, a 17.1 ppb increase in NO_2_ concentration over a 4-day period was associated with a 0.61% (95% confidence interval = 0.27%, 0.95%) increase in AD/ADRD ED visits. For PM_2.5_, a positive association with AD/ADRD ED visits was found only in New York (0.64%, 95% confidence interval = 0.26%, 1.01% per 6.3 µg/m^3^). Associations with warm-season ozone levels were null.

**Conclusions:**

Our results suggest AD/ADRD patients are vulnerable to short-term health effects of ambient air pollution and strategies to lower exposure may reduce morbidity.

**What this study adds**

Alzheimer’s disease (AD) and Alzheimer’s disease-related dementia (ADRD) have affected millions of individuals and placed substantial burdens on public health worldwide. Few studies have investigated the short-term association between air pollution exposure and AD/ADRD morbidity. We analyzed over 7.5 million records of AD/ADRD emergency department visits from five US states, and report positive associations for short-term exposures to NO_2_ and PM_2.5_. Our findings suggest that AD/ADRD patients could be more vulnerable to air pollution due to their higher prevalence of comorbid conditions.

## Introduction

Ambient air pollution causes significant harm to the environment and public health, leading to a high global disease burden.^1^ Large population-based studies have consistently shown that long and short-term exposures to air pollutants, including ambient fine particulate matter (PM ≤2.5 µm in aerodynamic diameter [PM_2.5_]), nitrogen dioxide (NO_2_), and ozone, were associated with various adverse health outcomes. Specifically, exposures to PM_2.5_ and NO_2_ have been linked to respiratory diseases, cardiovascular diseases, and neurological damage,^2–7^ while ozone exposure is associated with elevated risks of respiratory diseases and multiple causes of mortality.^8,9^

Dementias represent a group of symptoms affecting memory, thinking, and social abilities, with the majority of cases due to Alzheimer’s disease (AD) and its related dementias (ADRD).^10^ Dementia affects a large and growing number of elderly people in the United States. It was estimated that 5.7 million Americans had Alzheimer’s dementia in 2018,^11^ and the annual healthcare cost was estimated to be $355 billion in 2021.^12^ Previous research has shown that long-term exposure to air pollution is an emerging risk factor for dementia.^13–16^ For example, a cohort study conducted among US Medicare beneficiaries from 2000 to 2018 found that 5-year average PM_2.5_ and NO_2_ concentrations were associated with elevated incidences for both dementia and AD.^7^ Air pollution has also been associated with cognitive decline in elderly cohorts.^17^ Pathology studies also support the adverse effect of air pollution on brain structure and cognitive abilities.^18–21^

Despite the increasing interest in air pollution and dementia research, few studies have investigated the short-term association between air pollution exposure and AD/ADRD morbidity. AD/ADRD patients have a high prevalence of comorbid conditions, such as cerebrovascular diseases, kidney diseases, and diabetes,^22–24^ which have been shown to be exacerbated by short-term exposure to air pollution.^25,26^ Case studies have found that 20% of surveyed dementia patients living in the community experienced at least one emergency department (ED) visit in the past 3 months,^27^ while more than one-third of those in long-term care facilities had one or more ED visits in the past year.^28,29^ A recent analysis of the US Medicare database showed that the majority of dementia-related ED visits had higher Medicare payments, greater comorbidities, and higher return rates compared to those without dementia.^30^

To fill this knowledge gap regarding the potential impacts of short-term air pollution exposure on AD/ADRD morbidity, we analyzed hospital billing records among patients over 45 years old in five US states: California, Missouri, North Carolina, New Jersey, and New York. We conducted time-stratified case-crossover analyses to estimate short-term associations between exposure to three criteria air pollutants (PM_2.5_, NO_2_, warm-season ozone), and AD/ADRD, using ED visits as the morbidity outcome measure. This study aims to contribute to our understanding of the health effects of air pollution among AD/ADRD patients that may help support patients and their caregivers to reduce morbidity and improve care.

## Methods

### Emergency department visit, air pollution, and meteorology data

We obtained patient-level ED visits from hospital associations or state health departments in five US states: California (2005–2015), Missouri (2005–2015), North Carolina (2011–2015), New Jersey (2005–2015), and New York (2005–2015). The definition of an ED visit included patients seen in ED and either treated and discharged directly, or admitted to the hospital. ED records included admission date, age in years, self-reported patient’s residential ZIP code, and International Classification of Diseases diagnosis codes. We applied the condition algorithm from the Centers for Medicare and Medicaid Services to identify AD and ADRD ED visits using both primary and secondary diagnosis codes (Table S1; http://links.lww.com/EE/A214).^31^ Patients 45 years and older were included in the study.

Daily ambient air pollution data for PM_2.5_ (24-hour average), NO_2_ (1-hour maximum), and ozone (8-hour maximum) concentrations were estimated at a 1 km × 1 km spatial resolution in the United States from 2000 to 2016, using spatiotemporal ensemble models.^32–34^ Specifically, the ensemble-based models used over 100 predictors, integrated three machine learning algorithms (including a neural network, a random forest, and a gradient boosting machine), and achieved excellent model performance.^35–37^ Air pollution data were aggregated to the patient’s ZIP code level for each day by averaging the daily concentrations of all 1 km^2^ grid cells of which the centroids fell within the ZIP code boundary. Given that ozone is more readily formed in the warm season, and negatively correlated with NO_2_ and PM_2.5_ in the cold season, we restricted the analysis for ozone to the warm season (May to October).^38^

Daily maximum and minimum temperature in degrees Celsius, and water vapor pressure (a measure of humidity) in pascals at 1 km × 1 km spatial resolution were acquired from Daymet.^39^ Daily average temperature was calculated by taking the average of daily maximum and minimum temperature. The daily 1-km gridded Daymet data were aggregated to the 2010 ZIP code tabulation areas boundary by spatial averaging. Population sizes by age group in 2010 were obtained from Decennial Census 2010 datasets of the US Census Bureau.^40^

### Statistical analyses

To estimate state-specific and overall short-term effects of air pollutant concentrations on AD/ADRD-related ED visits, we applied a two-stage approach. In the first stage, state-specific associations were estimated using conditional logistic regression models with a case-crossover design. For each patient, we selected all days with the same day of the week in the same year and month of the ED visit day as control days. Controls are also matched by patient ZIP code. This case-crossover design automatically controls for individual-level time-invariant confounders (e.g., age, sex, race). In our primary analysis, we utilized an unconstrained distributed lagged model which simultaneously included same-day air pollutant concentrations (lag 0) and concentrations from the previous 3 days (lag 1, lag 2 and lag3) in the model. Each exposure (PM_2.5_, NO_2_, ozone) was fitted separately in the analyses. Patients 45 years and older were included in the analysis.

Our models also included several time-varying confounders. First, natural cubic splines on day of the year with 4 degrees of freedom (df) were used to control for within-month residual temporal trend. Second, we controlled for same-day and 3-day moving average of daily average temperature and dew-point temperature using natural cubic splines with 6 df for the all-year analyses, and 3 df for the warm-season only analyses. Finally, indicators of federal holidays were included. For each pollutant of interest, we reported the cumulative effect across exposure days from the distributed lag models by summing the four regression coefficients associated with exposure lags 0 to 3. To summarize the overall effect of a pollutant on AD/ADRD-related ED visits across states, in the second stage, state-specific log odds ratios were combined using inverse-variance weighting.

We also estimated associations between air pollutant concentrations and ED visits among non-AD/ADRD ED visits, to examine whether the AD/ADRD patient population is at higher risk compared to the non-AD/ADRD population of ED users. This analysis is restricted to those ages >75. Because of the large counts of non-AD/ADRD ED visits, the equivalent conditional Poisson model was used following the same the case-crossover design and confounder adjustment.^41^ Finally, we examined associations only among AD ED visits as a sensitivity analysis.

We implemented several sensitivity analyses to assess the robustness of results from our primary analysis by modeling the overall temporal trend using calendar date with varying degrees of freedom from 6 to 12 per year, and examining associations of air pollutant concentrations separately at different lags (lags 0 to 3), and a 4-day (lag 0 to 3) moving average. All data analyses were performed in R 4.0.2.^42^

## Results

Table 1 shows the number of AD and AD/ADRD ED visits in the study period by state and age group. Overall, our study included 1,595,783 AD ED visits and 6,119,274 AD/ADRD ED visits. Most of the AD ED visits (86.8%) or AD/ADRD ED visits (84.1%) were among patients 75 years and older. Table S2; http://links.lww.com/EE/A214 shows ED visit counts by case ascertainment (primary versus secondary diagnosis) and whether the patient was admitted or discharged. The majority of AD/ADRD ED visits were ascertained by secondary diagnosis codes (94.7%) and resulted in hospitalization (65.6%). Among ED visits with AD/ADRD as a secondary diagnosis, 15.7% had a primary diagnosis for cardiovascular disease and 11.5% had a primary diagnosis for respiratory disease (Table S3; http://links.lww.com/EE/A214). A larger proportion of AD ED visits (65.1%) or AD/ADRD ED visits (63.4%) were by female patients (Table S4; http://links.lww.com/EE/A214). The annual rates of AD/ADRD ED visits were 586.55 per 100,000 person-year for AD and 2,208.56 per 100,000 person-year for AD/ADRD. State-specific estimates are given in Table S5; http://links.lww.com/EE/A214.

**Table 1.** Total number of ED visits stratified by AD/ADRD status and age groups in five US states: California (2005–2015), Missouri (2005–2015), North Carolina (2011–2015), New Jersey (2005–2015), and New York (2005–2015).

State	Age group	AD	AD/ADRD	Non-AD/ADRD
California	Ages 45–64	14,627	97,107	28,545,677
	Ages 65–74	62,219	277,824	8,626,262
	Ages 75+	563,196	2,165,510	11,697,982
	All	640,042	2,540,441	48,869,921
Missouri	Ages 45–64	4,147	22,738	5,766,638
	Ages 65–74	16,484	58,581	1,781,156
	Ages 75+	116,995	364,822	2,298,349
	All	137,626	446,141	9,846,143
North Carolina	Ages 45–64	4,958	36,606	9,243,591
	Ages 65–74	21,877	105,966	3,020,506
	Ages 75+	140,221	569,906	3,411,259
	All	167,056	712,478	15,675,356
New Jersey	Ages 45–64	7,244	33,642	8,387,788
	Ages 65–74	24,891	83,479	2,391,053
	Ages 75+	219,708	661,078	3,442,414
	All	251,843	778,199	14,221,255
New York	Ages 45–64	11,684	66,083	18,466,530
	Ages 65–74	42,582	185,043	5,402,766
	Ages 75+	344,950	1,390,889	7,400,257
	All	399,216	1,642,015	31,269,553
5-state	All	1,595,783	6,119,274	119,882,228

Summary statistics of PM_2.5_, NO_2_, and warm-season ozone concentrations across the study period by states are shown in Table 2. The mean concentrations for PM_2.5_, NO_2,_ and warm-season ozone were 9.1 µg/m^3^, 17.9 ppb, and 43.8 ppb, respectively. The PM_2.5_ and warm-season ozone concentrations were similar across five states, while there was a larger variation in NO_2_. Specifically, the mean and interquartile range (IQR) of NO_2_ concentrations in Missouri (12.06 ppb, IQR = 8.40 ppb) and North Carolina (12.47 ppb, IQR = 8.20 ppb) were much lower than the 5-state average (17.94 ppb, IQR = 17.11 ppb), while the mean NO_2_ concentration in New Jersey (25.29 ppb) was much higher than the average.

**Table 2.** Mean, SD, and IQR of air pollutant level by states and average across all states.

State	PM_2.5_		NO_2_		Warm-season ozone	
	Mean (SD)	IQR	Mean (SD)	IQR	Mean (SD)	IQR
California	9.15 (7.42)	7.12	20.65 (14.19)	20.26	46.21 (13.20)	17.75
Missouri	9.80 (5.17)	6.39	12.06 (8.40)	8.84	43.95 (10.88)	14.66
North Carolina	9.68 (5.32)	6.41	12.47 (8.20)	9.50	43.56 (11.11)	15.09
New Jersey	9.73 (6.23)	7.09	25.29 (12.67)	18.01	43.25 (12.59)	17.44
New York	8.21 (5.56)	6.31	18.08 (14.65)	16.70	41.04 (11.00)	14.74
5-state	9.12 (6.26)	6.77	17.94 (13.40)	17.11	43.78 (12.10)	16.19

Units of air pollutant concentrations are NO_2_ (ppb), PM_2.5_ (µg/m^3^), and warm-season ozone (ppb).

**Figure 1.**
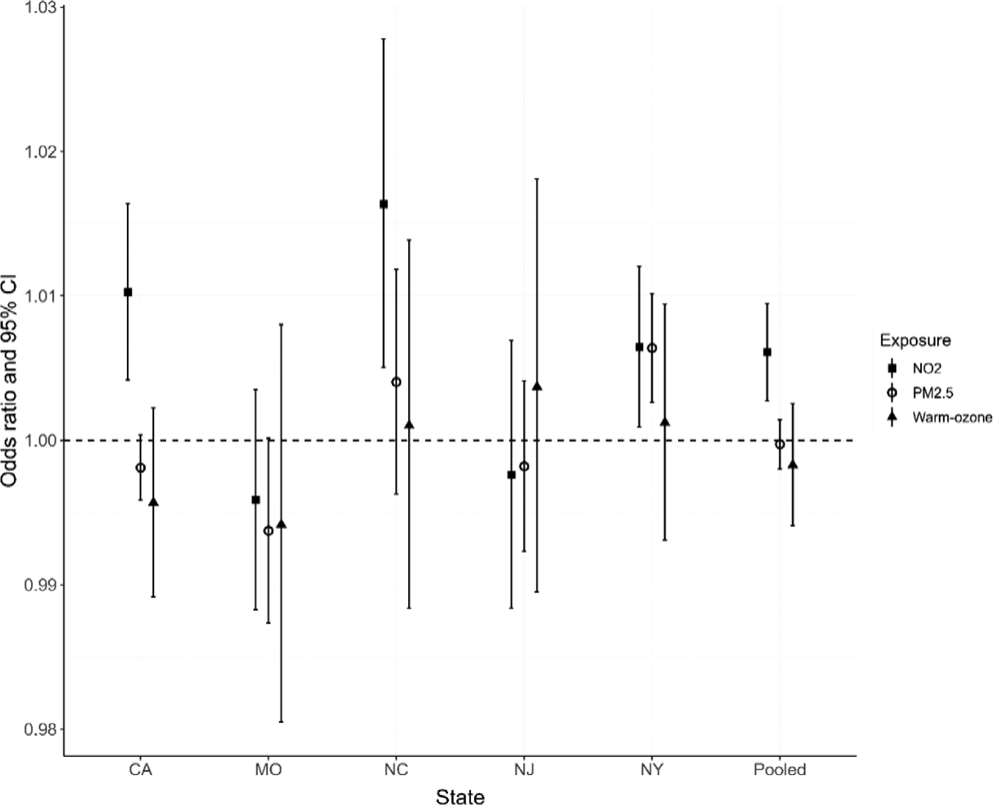
Four-day cumulative associations between AD/ADRD ED visits and an IQR increase of PM_2.5_, NO_2_, and warm-season (May to October) ozone in 5 states: California (CA), Missouri (MO), North Carolina (NC), New Jersey (NJ), and New York (NY). Cumulative associations were estimated from a distributed-lag model (lag 0 to lag 3). Pooled estimates were derived from inverse variance weighting. Odds ratios were adjusted by meteorology, holidays, and seasonality; time trends (year, month, day of week) were controlled automatically by the case-crossover design.

Figure 1 shows the 4-day cumulative state-specific and pooled associations of daily NO_2_, PM_2.5_, and warm-season ozone levels on AD/ADRD ED visits from distributed-lag models with exposure lags 0 to 3. From pooled results, an IQR increase in NO_2_ concentration was associated with a 0.61% (95% confidence interval [CI] = 0.27%, 0.95%) increase in AD/ADRD ED visits. Positive state-specific associations for NO_2_ were also found for California, North Carolina and New York, but not in Missouri and New Jersey. While no statistically significant association was found for PM_2.5_ (−0.03%, 95% CI = −0.20%, 0.14%) and warm-season ozone (−0.27%, 95% CI = −0.59%, 0.25%) in pooled analysis, a positive association was found between PM_2.5_ and AD/ADRD ED visits in New York (0.64%, 95% CI = 0.26%, 1.01%). When the outcome was restricted to only AD ED visits, the magnitude of associations for NO_2_ were similar to the AD/ADRD analysis but these estimates had greater confidence intervals due to a smaller sample size (Figure S1; http://links.lww.com/EE/A214).

Lag-specific associations from the distributed lag models are shown in Figure S2; http://links.lww.com/EE/A214. From pooled results across states, positive associations for same-day (lag 0) were found for NO_2_ (0.62%, 95% CI = 0.38%, 0.87%) and PM_2.5_ (0.25%, 95% CI = 0.09%, 0.40%) when controlling on exposure of other lag days (lag1, lag2, lag3), while no statistically significant lag-specific association was found for warm-season ozone (lag 0, 0.06%, 95% CI = −0.27%, 0.39%). As a sensitivity analysis, results from models that included only one lagged exposure at a time in the model are given in Figure S3; http://links.lww.com/EE/A214, and the positive associations with same-day NO_2_ and PM_2.5_ remain.

To explore whether the short-term effects of air pollution on ED visits were stronger for people with AD/ADRD, we compared the results from distributed-lag models to all other non-AD/ADRD ED visits (Figure S4; http://links.lww.com/EE/A214). Because most of the AD/ADRD patients are 75 years and older, we restricted the comparison only within this age group (75+ years). Overall, the cumulative effect of NO_2_ (lag 0 to lag 3) was higher for AD/ADRD ED visits (0.49%, 95% CI = 0.12%, 0.86%) compared to non-AD/ADRD ED visits (−0.0.06%, 95% CI = −0.43%, 0.10%), while no associations for PM_2.5_ and warm-season ozone were observed for either group.

Finally, we found that the estimated odds ratios in primary analyses were robust with respect to df for temporal splines. Numerical values of all estimated odds ratios and the corresponding 95% confidence intervals are provided in Tables S6 and S7; http://links.lww.com/EE/A214.

## Discussion

This study analyzed over 7.5 million AD/ADRD ED visits and their short-term associations with three criteria air pollutants: PM_2.5_, NO_2_, and warm-season ozone. For short-term cumulative effects over lag 0 to lag 3 days, we found that NO_2_ levels were significantly associated with increased AD/ADRD ED visits in multiple US states, while no 4-day pooled association was found for PM_2.5_ and warm-season ozone. Pooled associations were strongest for NO_2_ when considering same-day exposure levels. Same-day PM_2.5_ levels were also significantly associated with AD/ADRD ED visits in the pooled model, which was driven by New York data. For NO_2,_ the positive association with AD/ADRD ED visits was also stronger in comparison to non-AD/ADRD ED visits, which suggests that AD/ADRD patients may be more vulnerable to NO_2_ exposure than the general population.

Ambient NO_2_ is mainly emitted from mobile sources and industrial processes in urban areas. Previous studies have shown that short-term NO_2_ exposure was associated with the ED visits and hospitalizations of various diseases, as well as with mortality.^43–47^ Those studies also found that the associations were strongest for same-day NO_2_ exposure compared to longer-lagged exposures. AD/ADRD patients generally have a higher prevalence of cardiorespiratory diseases,^22,48^ which may explain the observed associations with NO_2_ due to exacerbations of these comorbidities.

Results for PM_2.5_ were mixed compared to NO_2_ and we observed heterogeneity in associations between states and between different lag days. A study conducted in Seoul reported a positive association between PM_2.5_ and emergency hospital admissions, and such association was strongest for same-day exposure and diminished when using lag 1, 2, or 3 day exposure,^49^ which was in agreement with our results for New York state. We observed several negative associations between PM_2.5_ and AD/ADRD ED visits in Missouri and New Jersey among those ages 75 or above. One possible explanation may be changes in behaviors on high air pollution days. For example, susceptible populations may have less exposure to ambient air pollution by staying indoors.

We did not find an association between daily ozone levels and AD/ADRD ED visits in the warm season. The null association was consistent in both distributed lag models and single lag models. While studies showed that ambient ozone exposure was associated with asthma and reduction of lung function among children, many studies have reported a null association between short-term ozone exposure and other respiratory outcomes.^50–52^ Therefore, our results and previous studies suggest that the short-term ozone exposures may not be a risk factor for the elderly AD/ADRD patient population.

The heterogeneity in associations across different states is notable. Many factors could contribute to the heterogeneity including the difference in population composition, geographical and climatic factors, and differences in PM_2.5_ composition. Particularly, our state-wide analyses included both urban and rural regions. Another explanation may be statistical power; the two largest states (California and New York) showed statistically significant positive associations.

In our analysis, the odds ratios for NO_2_ and AD/ADRD ED visits were larger compared to those for non-AD/ADRD ED visits in CA, NC, and NY. This provides suggestive evidence that the AD/ADRD patient population may be more at-risk to short-term exposure to ambient air pollution. The excessive risk might be attributed to the higher prevalence and more severe of comorbid conditions,^24,53^ as well as challenges in the management of chronic diseases due to impaired cognitive and physical functions.^54,55^ More comprehensive analyses are needed to investigate this hypothesis. A potential direction could be restricting the AD/ADRD and non-AD/ADRD ED visits to specific co-morbid conditions and examine whether the difference in association still persists.

This study has several strengths. First, our study accounted for multiple air pollutants that were estimated from ensemble-based models on a fine spatial scale, providing complete coverage and higher accuracy than using the monitor data only. Additionally, we tried to minimize the exposure heterogeneity due to spatial variation in ambient exposure levels by assigning ZIP code level exposures. Second, the number of AD/ADRD ED visits was large compared to other similar studies. Our assessment of morbidity included both ED patients treated and discharged directly from the ED and those admitted as inpatients through the ED, making the analysis comprehensive in terms of capturing AD/ADRD morbidity. The five states selected in this study also represented different geographic and climate regions in United States. Hence our study could provide more accurate risk estimates and more generalizable conclusions.

There are also some limitations in this study. First, the AD/ADRD outcome was defined based on International Classification of Diseases codes from billing records that are less accurate than medical records and adjudicated outcomes from prospective cohort studies. Second, we used temporal variations in outdoor air pollution levels as proxy of the variations in personal exposure levels. While this could introduce measurement error, we note that previous studies have shown that the effect of this kind of measurement errors often lead to attenuated estimated relative risks.^56–59^

## Conclusions

We observed a positive association between daily NO_2_ levels and AD/ADRD ED visits, while the association for PM_2.5_ levels was mixed, and no association was found for warm-season ozone. AD/ADRD patients could be more vulnerable to air pollution due to their higher prevalence of comorbid conditions.

## Conflicts of interest statement

The authors declare that they have no conflicts of interest with regard to the content of this report.

## ACKNOWLEDGMENTS

The data used to produce this publication were acquired from the: California Office of Statewide Health Planning and Development (years 2005–2016); the Missouri Department of Health and Senior Services (years 2005–2018); the North Carolina Hospital (inpatient, ambulatory surgery/outpatient, emergency room) Discharge Database (Truven Health Analytics, years 2011–2017) from the Cecil G. Sheps Center for Health Services Research and the North Carolina Division of Health Service Regulation; the Center for Health Statistics & Informatics of the New Jersey Department of Health, Trenton, NJ (years 2005–2016); and the Statewide Planning and Research Cooperative System of the New York State Department of Health (years 2005–2016). The contents of this publication including data analysis, interpretation, conclusions derived, and the views expressed herein are solely those of the authors and do not represent the conclusions or official views of data sources listed above. The data sources, their employees, officers, and agents make no representation, warranty or guarantee as to the accuracy, completeness, currency, or suitability of the information provided here.

## Supplementary Material


