# Supplementary material for: Short-term associations between ambient air pollution and emergency department visits for Alzheimer’s disease and related dementias
Source: Environ Epidemiol. 2022 Dec 22;7(1):e237. doi: 10.1097/EE9.0000000000000237 (PMC9915954; doi:10.1097/EE9.0000000000000237)

**Supplementary Materials for**

**Short-term Associations between Ambient Air Pollution and Emergency Department Visits for Alzheimer’s Disease and Related Dementias**

Haisu Zhang^1^, Liuhua Shi^1^, Stefanie T. Ebelt^1^, Rohan R. D’Souza^2^, Joel D. Schwartz,^3^ Noah Scovronick^1^, Howard H. Chang^1,2,*^

^1^Gangarosa Department of Environmental Health, Rollins School of Public Health, Emory University, Atlanta GA, USA

^2^Department of Biostatistics and Bioinformatics, Rollins School of Public Health, Emory University, Atlanta GA, USA

^3^Department of Environmental Health, Harvard T.H. Chan School of Public Health, Boston, MA, USA

*Corresponding author: Howard H Chang ([howard.chang@emory.edu](mailto:howard.chang@emory.edu)), 1518 Clifton Rd., NE,

Atlanta , GA 30322. Telephone (404) 712-4627

**Table S1** ICD codes used for identifying AD/ADRD ED visits, as defined by the Centers for Medicare and Medicaid Services. Both primary and secondary diagnosis codes considered for identifying visit of interest.

| ICD 9^th^ revision  (records prior to October 1, 2015) | ICD 10^th^ revision |
| --- | --- |
| AD | |
| 331.0 | G30.0, G30.1, G30.8, G30.9 |
| ADRD | |
| 331.11, 331.19, 331.2, 331.7, 290.0, 290.10, 290.11, 290.12, 290.13, 290.20, 290.21, 290.3, 290.40, 290.41, 290.42, 290.43, 294.0, 294.10, 294.11, 294.20, 294.21, 294.8, 797 | F01.50, F01.51, F02.80, F02.81, F03.90, F03.91, F04, G13.8, F05, F06.1, F06.8G31.1, G31.2, G31.01, G31.09, G94, R41.81, R54 |

**Table S2** Total number of AD/ADRD ED visits in five US states: California (CA 2005-2016), Missouri (MO 2005 - 2018), North Carolina (NC 2011 - 2017), New Jersey (NJ 2005 - 2016), and New York (NY 2005 - 2016) stratified (1) primary versus secondary diagnosis and (2) whether the ED patient was discharged directly or admitted to hospital.

| State | Diagnosis | AD/ADRD  ED Visits | Discharged | Admitted to Hospital |
| --- | --- | --- | --- | --- |
| CA | Primary | 106,716 | 66,243 (62.07%) | 40,473 (37.93%) |
|  | Secondary | 2,433,725 | 838,680 (34.46%) | 1,595,045 (65.54%) |
|  | All | 2,540,441 | 904,923 (35.62%) | 1,635,518 (64.38%) |
| MO | Primary | 28,756 | 13,112 (45.60%) | 15,644 (54.40%) |
|  | Secondary | 417,385 | 119,525 (28.64%) | 297,860 (71.36%) |
|  | All | 446,141 | 132,637 (29.73%) | 313,504 (70.27%) |
| NC | Primary | 30,113 | 22,595 (75.03%) | 7,518 (24.97%) |
|  | Secondary | 682,365 | 447,541 (65.59%) | 234,824 (34.41%) |
|  | All | 712,478 | 470,136 (65.99%) | 242,342 (34.01%) |
| NJ | Primary | 50,110 | 26,127 (52.14%) | 23,983 (47.86%) |
|  | Secondary | 728,089 | 200,563 (27.55%) | 527,526 (72.45%) |
|  | All | 778,199 | 226,690 (29.13%) | 551,509 (70.87%) |
| NY | Primary | 106,458 | 48,736 (45.78%) | 57,722 (54.22%) |
|  | Secondary | 1,535,557 | 320,461 (20.87%) | 1,215,096 (79.13%) |
|  | All | 1,642,015 | 369,197 (22.48%) | 1,272,818 (77.52%) |
| All states | Primary | 322,153 | 176,813 (54.88%) | 145,340 (45.12%) |
|  | Secondary | 5,797,121 | 1,926,770 (33.24%) | 3,870,351 (66.76%) |
|  | All | 6,119,274 | 2,103,583 (34.38%) | 4,015,691 (65.62%) |

**Table S3.** Counts of ED visits for select primary diagnoses among ED visits with AD/ADRD ascertained with secondary diagnosis codes.

| **Diseases** | **Diagnosis Codes** | | **State-specific ED visit Counts** | | | | | **Overall Counts** |
| --- | --- | --- | --- | --- | --- | --- | --- | --- |
|  | **ICD 9** | **ICD 10** | **CA** | **MO** | **NC** | **NJ** | **NY** |  |
| **Cardiovascular Diseases** | 390-459 | I00-I99 | 356,046 | 69,663 | 111,403 | 127,127 | 255,076 | 919,315 |
| **Stroke** | 433-437 | G45, I64-I67 | 82,933 | 16,221 | 27,649 | 27,665 | 53,907 | 208,375 |
| **Hypertension** | 401-405 | I10-I15 | 21,230 | 3,997 | 6,793 | 8,496 | 15,002 | 55,518 |
| **Congestive heart failure** | 428 | I42, I50, I 51 | 74,329 | 16,005 | 24,903 | 27,196 | 57,375 | 199,808 |
| **Diabetes** | 249, 250 | E08-E13 | 33,309 | 5,254 | 9,369 | 11,177 | 27,084 | 86,193 |
| **Respiratory Diseases** | 460-519 | J00-J99 | 282,967 | 55,137 | 79,651 | 83,278 | 172,233 | 673,266 |
| **COPD** | 491-492, 496 | J41-J44 | 30,608 | 6,906 | 10,867 | 13,896 | 24,704 | 86,981 |
| **Kidney diseases** | 580-590 | N00-N19 | 61,342 | 12,772 | 27,011 | 19,765 | 40,405 | 161,295 |
|  |  |  |  |  |  |  |  |  |
| **Secondary diagnosis of AD/ADRD** | See Table S1 | | 2,450,977 | 417,417 | 707,538 | 739,615 | 1,548,406 | 5,863,953 |

**Table S4.** Total number of AD/ADRD ED visits and the subset of AD ED visits stratified by status and sex in five US states: California (2005 - 2015), Missouri (2005 - 2015), North Carolina (2011 - 2015), New Jersey (2005 - 2015), and New York (2005 - 2015).

| State | Sex | AD | AD/ADRD |
| --- | --- | --- | --- |
| California | Male | 230,526 | 958,470 |
|  | Female | 409,454 | 1,581,802 |
|  | All | 640,042 | 2,540,441 |
| Missouri | Male | 48,337 | 163,588 |
|  | Female | 89,289 | 282,553 |
|  | All | 137,626 | 446,141 |
| North Carolina | Male | 54,629 | 251,164 |
|  | Female | 112,423 | 461,307 |
|  | All | 167,056 | 712,478 |
| New Jersey | Male | 86,564 | 280,429 |
|  | Female | 165,279 | 497,770 |
|  | All | 251,843 | 778,199 |
| New York | Male | 136,501 | 585,506 |
|  | Female | 262,712 | 1,056,501 |
|  | All | 399,216 | 1,642,015 |
| All | Male | 556,557 | 2,239,157 |
|  | Female | 1,039,157 | 3,879,933 |
|  | All | 1,595,783 | 6,119,274 |

*Some patients had missing sex; so sex-specific numbers may not add up to the total.

**Table S5.** Total number and estimated annual rates of AD/ADRD ED visits and the AD ED visits subset stratified by age groups in five US states: California (2005 - 2015), Missouri (2005 - 2015), North Carolina (2011 - 2015), New Jersey (2005 - 2015), and New York (2005 - 2015).

| State | Age Group | AD | | AD/ADRD | | Population in 2010 |
| --- | --- | --- | --- | --- | --- | --- |
|  |  | ED visit count* | Est. rate  per 100,000 PY^+^ | ED visit count* | Est. rate  per 100,000 PY^+^ |  |
| California | Ages 45-64 | 14,627 | 14.32 | 97,107 | 95.04 | 9,288,864 |
|  | Ages 65-74 | 62,219 | 248.59 | 277,824 | 1,110.02 | 2,275,336 |
|  | Ages 75+ | 563,196 | 2,597.41 | 2,165,510 | 9,987.15 | 1,971,178 |
|  | All | 640,042 | 429.88 | 2,540,441 | 1,706.26 | 13,535,378 |
| Missouri | Ages 45-64 | 4,147 | 23.39 | 22,738 | 128.24 | 1,611,850 |
|  | Ages 65-74 | 16,484 | 332.65 | 58,581 | 1,182.17 | 450,490 |
|  | Ages 75+ | 116,995 | 2,742.60 | 364,822 | 8,552.16 | 387,804 |
|  | All | 137,626 | 510.64 | 446,141 | 1,655.34 | 2,450,144 |
| North Carolina | Ages 45-64 | 11,684 | 93.20 | 66,083 | 527.10 | 2,507,407 |
|  | Ages 65-74 | 42,582 | 1,220.87 | 185,043 | 5,305.38 | 697,567 |
|  | Ages 75+ | 344,950 | 12,858.99 | 1,390,889 | 51,849.32 | 536,512 |
|  | All | 399,216 | 2,134.00 | 1,642,015 | 8,777.34 | 3,741,486 |
| New Jersey | Ages 45-64 | 4,958 | 18.58 | 36,606 | 137.21 | 2,425,361 |
|  | Ages 65-74 | 21,877 | 325.27 | 105,966 | 1,575.52 | 611,434 |
|  | Ages 75+ | 140,221 | 2,218.63 | 569,906 | 9,017.29 | 574,559 |
|  | All | 167,056 | 420.53 | 712,478 | 1,793.53 | 3,611,354 |
| New York | Ages 45-64 | 7,244 | 12.71 | 33,642 | 59.01 | 5,182,359 |
|  | Ages 65-74 | 24,891 | 166.31 | 83,479 | 557.77 | 1,360,602 |
|  | Ages 75+ | 219,708 | 1,588.55 | 661,078 | 4,779.77 | 1,257,341 |
|  | All | 251,843 | 293.51 | 778,199 | 906.96 | 7,800,302 |
| All | All | 1,595,783 | 586.55 | 6,119,274 | 2,208.56 | 31,138,664 |

*ED visit count across the study period.

^+^ Estimated rate of ED visits per 100,000 person-year. Rates for all state were calculated based on annual average ED visit counts of each state.

**Table S6**. Relative risks estimates of association between ED visits for AD/ADRD and per IQR increase of PM2.5, NO2, warm-season ozone (lag 0 to lag 3 and lag 0-3 cumulative effect) from a distributed-lag model. Pooled estimates were derived from inverse variance weighting.

| Outcome | Pollutant | Lag days | State | | | | | |
| --- | --- | --- | --- | --- | --- | --- | --- | --- |
|  |  |  | CA | MO | NC | NJ | NY | State Pooled |
| ADRD | NO_2_ | Lag 0 | 1.0062 (1.0011, 1.0112) | 1.0017 (0.9965, 1.0069) | 1.0091 (1.0014, 1.0168) | 1.0064 (1.0002, 1.0127) | 1.0066 (1.0028, 1.0103) | 1.0062 (1.0038, 1.0087) |
|  |  | Lag 1 | 1.0036 (0.9981, 1.0091) | 0.9983 (0.9927, 1.0039) | 1.0012 (0.9932, 1.0094) | 0.9914 (0.9850, 0.9979) | 1.0001 (0.9962, 1.0041) | 0.9995 (0.9969, 1.0021) |
|  |  | Lag 2 | 1.0000 (0.9946, 1.0056) | 0.9980 (0.9924, 1.0036) | 0.9981 (0.9900, 1.0063) | 1.0016 (0.9952, 1.0081) | 0.9984 (0.9945, 1.0024) | 0.9993 (0.9967, 1.0019) |
|  |  | Lag 3 | 1.0004 (0.9958, 1.0051) | 0.9979 (0.9928, 1.0030) | 1.0079 (1.0008, 1.0150) | 0.9981 (0.9923, 1.0040) | 1.0013 (0.9978, 1.0049) | 1.0006 (0.9983, 1.0029) |
|  |  | Lag 0-3 | 1.0103 (1.0042, 1.0164) | 0.9959 (0.9883, 1.0035) | 1.0164 (1.0051, 1.0278) | 0.9976 (0.9884, 1.0069) | 1.0065 (1.0009, 1.0120) | 1.0061 (1.0027, 1.0095) |
|  |  |  |  |  |  |  |  |  |
|  | PM_2.5_ | Lag 0 | 1.0008 (0.9983, 1.0033) | 1.0016 (0.9956, 1.0077) | 0.9982 (0.9919, 1.0044) | 1.0004 (0.9960, 1.0048) | 1.0067 (1.0039, 1.0095) | 1.0025 (1.0009, 1.0040) |
|  |  | Lag 1 | 1.0004 (0.9972, 1.0036) | 0.9961 (0.9889, 1.0033) | 0.9968 (0.9897, 1.0039) | 0.9998 (0.9950, 1.0046) | 0.9982 (0.9952, 1.0013) | 0.9990 (0.9971, 1.0009) |
|  |  | Lag 2 | 0.9988 (0.9956, 1.0021) | 0.9960 (0.9890, 1.0032) | 1.0043 (0.9973, 1.0114) | 0.9974 (0.9928, 1.0021) | 1.0016 (0.9986, 1.0046) | 0.9998 (0.9979, 1.0016) |
|  |  | Lag 3 | 0.9981 (0.9956, 1.0005) | 1.0000 (0.9940, 1.0060) | 1.0048 (0.9989, 1.0106) | 1.0006 (0.9965, 1.0047) | 0.9998 (0.9973, 1.0024) | 0.9996 (0.9980, 1.0011) |
|  |  | Lag 0-3 | 0.9981 (0.9958, 1.0004) | 0.9937 (0.9873, 1.0002) | 1.0040 (0.9963, 1.0118) | 0.9982 (0.9923, 1.0041) | 1.0064 (1.0026, 1.0101) | 0.9997 (0.9980, 1.0014) |
|  |  |  |  |  |  |  |  |  |
|  | Ozone | Lag 0 | 1.0019 (0.9962, 1.0076) | 1.0024 (0.9914, 1.0134) | 1.0072 (0.9974, 1.0170) | 0.9994 (0.9901, 1.0088) | 0.9969 (0.9913, 1.0025) | 1.0006 (0.9973, 1.0039) |
|  |  | Lag 1 | 0.9984 (0.9921, 1.0046) | 0.9964 (0.9861, 1.0069) | 0.9953 (0.9856, 1.0051) | 1.0061 (0.9977, 1.0147) | 1.0007 (0.9955, 1.0059) | 0.9999 (0.9967, 1.0032) |
|  |  | Lag 2 | 0.9949 (0.9886, 1.0011) | 0.9970 (0.9865, 1.0077) | 1.0067 (0.9968, 1.0168) | 0.9966 (0.9882, 1.0051) | 1.0003 (0.9951, 1.0054) | 0.9984 (0.9952, 1.0017) |
|  |  | Lag 3 | 1.0006 (0.9953, 1.0060) | 0.9983 (0.9892, 1.0076) | 0.9919 (0.9836, 1.0002) | 1.0016 (0.9940, 1.0092) | 1.0034 (0.9988, 1.0081) | 1.0006 (0.9977, 1.0034) |
|  |  | Lag 0-3 | 0.9957 (0.9892, 1.0022) | 0.9942 (0.9805, 1.0080) | 1.0010 (0.9884, 1.0139) | 1.0037 (0.9895, 1.0181) | 1.0012 (0.9931, 1.0094) | 0.9983 (0.9941, 1.0025) |
|  |  |  |  |  |  |  |  |  |
| AD | NO_2_ | Lag 0 | 1.0038 (0.9939, 1.0138) | 1.0106 (1.0013, 1.0200) | 1.0239 (1.0080, 1.0400) | 1.0098 (0.9990, 1.0208) | 1.0125 (1.0049, 1.0201) | 1.0104 (1.0057, 1.0152) |
|  |  | Lag 1 | 1.0097 (0.9989, 1.0205) | 0.9939 (0.9840, 1.0040) | 1.0049 (0.9884, 1.0216) | 0.9830 (0.9718, 0.9943) | 0.9950 (0.9871, 1.0029) | 0.9965 (0.9915, 1.0016) |
|  |  | Lag 2 | 0.9895 (0.9789, 1.0003) | 0.9944 (0.9844, 1.0044) | 0.9889 (0.9725, 1.0055) | 1.0000 (0.9888, 1.0113) | 0.9982 (0.9903, 1.0061) | 0.9953 (0.9904, 1.0004) |
|  |  | Lag 3 | 1.0112 (1.0019, 1.0205) | 1.0005 (0.9914, 1.0098) | 1.0004 (0.9861, 1.0149) | 0.9978 (0.9876, 1.0080) | 0.9990 (0.9918, 1.0062) | 1.0024 (0.9979, 1.0068) |
|  |  | Lag 0-3 | 1.0141 (1.0022, 1.0262) | 0.9994 (0.9857, 1.0132) | 1.0178 (0.9948, 1.0413) | 0.9905 (0.9746, 1.0066) | 1.0045 (0.9935, 1.0157) | 1.0055 (0.9991, 1.0120) |
|  |  |  |  |  |  |  |  |  |
|  | PM_2.5_ | Lag 0 | 1.0019 (0.9970, 1.0068) | 1.0040 (0.9932, 1.0148) | 1.0033 (0.9905, 1.0163) | 0.9971 (0.9896, 1.0046) | 1.0087 (1.0032, 1.0142) | 1.0033 (1.0002, 1.0063) |
|  |  | Lag 1 | 0.9989 (0.9925, 1.0052) | 0.9892 (0.9766, 1.0020) | 0.9951 (0.9807, 1.0097) | 0.9967 (0.9886, 1.0050) | 0.9927 (0.9868, 0.9987) | 0.9954 (0.9919, 0.9990) |
|  |  | Lag 2 | 1.0002 (0.9939, 1.0065) | 0.9968 (0.9843, 1.0095) | 0.9914 (0.9774, 1.0057) | 0.9930 (0.9851, 1.0009) | 1.0013 (0.9955, 1.0071) | 0.9983 (0.9948, 1.0018) |
|  |  | Lag 3 | 0.9974 (0.9925, 1.0022) | 0.9995 (0.9889, 1.0101) | 1.0002 (0.9884, 1.0122) | 1.0049 (0.9979, 1.0119) | 0.9977 (0.9927, 1.0029) | 0.9992 (0.9962, 1.0021) |
|  |  | Lag 0-3 | 0.9983 (0.9939, 1.0027) | 0.9894 (0.9782, 1.0007) | 0.9901 (0.9744, 1.0060) | 0.9916 (0.9816, 1.0018) | 1.0003 (0.9930, 1.0078) | 0.9969 (0.9937, 1.0002) |
|  |  |  |  |  |  |  |  |  |
|  | Ozone | Lag 0 | 1.0100 (0.9987, 1.0214) | 0.9922 (0.9728, 1.0121) | 1.0078 (0.9878, 1.0283) | 0.9954 (0.9796, 1.0115) | 0.9945 (0.9836, 1.0056) | 1.0010 (0.9947, 1.0074) |
|  |  | Lag 1 | 0.9964 (0.9842, 1.0088) | 1.0096 (0.9906, 1.0289) | 1.0040 (0.9838, 1.0246) | 1.0043 (0.9899, 1.0189) | 0.9998 (0.9895, 1.0101) | 1.0011 (0.9948, 1.0073) |
|  |  | Lag 2 | 0.9899 (0.9777, 1.0022) | 0.9827 (0.9639, 1.0018) | 1.0047 (0.9842, 1.0256) | 0.9935 (0.9792, 1.0081) | 0.9994 (0.9893, 1.0096) | 0.9944 (0.9882, 1.0006) |
|  |  | Lag 3 | 1.0007 (0.9902, 1.0113) | 1.0019 (0.9854, 1.0187) | 0.9847 (0.9677, 1.0019) | 1.0015 (0.9886, 1.0146) | 1.0040 (0.9949, 1.0132) | 1.0005 (0.9951, 1.0060) |
|  |  | Lag 0-3 | 0.9968 (0.9840, 1.0098) | 0.9862 (0.9621, 1.0110) | 1.0010 (0.9749, 1.0278) | 0.9947 (0.9706, 1.0194) | 0.9977 (0.9816, 1.0140) | 0.9961 (0.9880, 1.0043) |

**Table S7**. Relative risk estimates of association between ED visits for AD/ADRD and per IQR increase of PM_2.5_, NO2, warm-season ozone (lag 0 to lag 3 and lag 0-3 average). Different lagged exposures were fitted separately with conditional logistic regression models. Pooled estimates were derived from inverse variance weighting.

| Outcome | Pollutant | Lag days | State | | | | | |
| --- | --- | --- | --- | --- | --- | --- | --- | --- |
|  |  |  | CA | MO | NC | NJ | NY | State Pooled |
| ADRD | NO_2_ | Lag 0 | 1.0081 (1.0038, 1.0125) | 1.0005 (0.9957, 1.0054) | 1.0093 (1.0022, 1.0164) | 1.0035 (0.9977, 1.0093) | 1.0065 (1.0030, 1.0100) | 1.0062 (1.0040, 1.0084) |
|  |  | Lag 1 | 1.0067 (1.0026, 1.0109) | 0.9978 (0.9930, 1.0027) | 1.0036 (0.9968, 1.0105) | 0.9940 (0.9883, 0.9997) | 1.0017 (0.9983, 1.0052) | 1.0019 (0.9998, 1.0041) |
|  |  | Lag 2 | 1.0037 (0.9995, 1.0080) | 0.9967 (0.9919, 1.0016) | 1.0020 (0.9950, 1.0091) | 0.9984 (0.9927, 1.0041) | 0.9992 (0.9958, 1.0027) | 1.0004 (0.9982, 1.0026) |
|  |  | Lag 3 | 1.0016 (0.9975, 1.0058) | 0.9971 (0.9923, 1.0019) | 1.0071 (1.0006, 1.0138) | 0.9988 (0.9933, 1.0043) | 1.0009 (0.9975, 1.0042) | 1.0008 (0.9987, 1.0029) |
|  |  | Lag 0-3 moving average | 1.0099 (1.0040, 1.0159) | 0.9951 (0.9876, 1.0027) | 1.0144 (1.0032, 1.0256) | 0.9964 (0.9873, 1.0056) | 1.0050 (0.9995, 1.0105) | 1.0051 (1.0018, 1.0085) |
|  |  |  |  |  |  |  |  |  |
|  | PM_2.5_ | Lag 0 | 1.0003 (0.9985, 1.0020) | 0.9981 (0.9932, 1.0030) | 0.9977 (0.9922, 1.0031) | 1.0001 (0.9962, 1.0041) | 1.0061 (1.0036, 1.0086) | 1.0014 (1.0001, 1.0026) |
|  |  | Lag 1 | 0.9994 (0.9976, 1.0011) | 0.9945 (0.9896, 0.9994) | 0.9988 (0.9933, 1.0043) | 0.9988 (0.9949, 1.0027) | 1.0016 (0.9991, 1.0040) | 0.9995 (0.9983, 1.0008) |
|  |  | Lag 2 | 0.9981 (0.9963, 0.9998) | 0.9942 (0.9895, 0.9989) | 1.0053 (1.0000, 1.0106) | 0.9976 (0.9938, 1.0014) | 1.0014 (0.9990, 1.0038) | 0.9990 (0.9978, 1.0002) |
|  |  | Lag 3 | 0.9976 (0.9958, 0.9993) | 0.9966 (0.9919, 1.0013) | 1.0063 (1.0014, 1.0113) | 0.9996 (0.9959, 1.0033) | 1.0008 (0.9984, 1.0031) | 0.9991 (0.9978, 1.0003) |
|  |  | Lag 0-3 moving average | 0.9982 (0.9960, 1.0004) | 0.9928 (0.9866, 0.9991) | 1.0048 (0.9971, 1.0125) | 0.9977 (0.9919, 1.0036) | 1.0054 (1.0018, 1.0091) | 0.9995 (0.9978, 1.0012) |
|  |  |  |  |  |  |  |  |  |
|  | Ozone | Lag 0 | 0.9996 (0.9948, 1.0044) | 0.9997 (0.9899, 1.0096) | 1.0056 (0.9968, 1.0144) | 1.0013 (0.9925, 1.0103) | 0.9972 (0.9920, 1.0024) | 0.9997 (0.9968, 1.0027) |
|  |  | Lag 1 | 0.9967 (0.9921, 1.0013) | 0.9962 (0.9873, 1.0051) | 1.0005 (0.9923, 1.0087) | 1.0051 (0.9973, 1.0130) | 0.9994 (0.9948, 1.0041) | 0.9990 (0.9963, 1.0017) |
|  |  | Lag 2 | 0.9948 (0.9901, 0.9994) | 0.9956 (0.9864, 1.0048) | 1.0029 (0.9944, 1.0115) | 0.9981 (0.9899, 1.0062) | 1.0010 (0.9962, 1.0058) | 0.9980 (0.9952, 1.0007) |
|  |  | Lag 3 | 0.9980 (0.9934, 1.0026) | 0.9974 (0.9889, 1.0060) | 0.9944 (0.9868, 1.0021) | 1.0003 (0.9929, 1.0077) | 1.0034 (0.9989, 1.0079) | 0.9996 (0.9969, 1.0022) |
|  |  | Lag 0-3 moving average | 0.9947 (0.9883, 1.0012) | 0.9935 (0.9800, 1.0073) | 1.0010 (0.9885, 1.0138) | 1.0039 (0.9898, 1.0182) | 1.0014 (0.9933, 1.0095) | 0.9978 (0.9937, 1.0020) |
|  |  |  |  |  |  |  |  |  |
| AD | NO_2_ | Lag 0 | 1.0074 (0.9989, 1.0160) | 1.0075 (0.9987, 1.0163) | 1.0249 (1.0103, 1.0398) | 1.0039 (0.9938, 1.0140) | 1.0104 (1.0034, 1.0175) | 1.0089 (1.0046, 1.0132) |
|  |  | Lag 1 | 1.0081 (0.9999, 1.0163) | 0.9953 (0.9866, 1.0041) | 1.0094 (0.9954, 1.0237) | 0.9860 (0.9762, 0.9960) | 0.9985 (0.9916, 1.0054) | 0.9994 (0.9952, 1.0035) |
|  |  | Lag 2 | 1.0008 (0.9925, 1.0091) | 0.9933 (0.9846, 1.0021) | 0.9925 (0.9784, 1.0069) | 0.9940 (0.9842, 1.0039) | 0.9968 (0.9900, 1.0037) | 0.9968 (0.9926, 1.0010) |
|  |  | Lag 3 | 1.0083 (1.0002, 1.0165) | 0.9985 (0.9900, 1.0072) | 0.9964 (0.9832, 1.0098) | 0.9980 (0.9885, 1.0075) | 0.9987 (0.9920, 1.0054) | 1.0013 (0.9972, 1.0053) |
|  |  | Lag 0-3 moving average | 1.0122 (1.0005, 1.0241) | 0.9965 (0.9830, 1.0102) | 1.0141 (0.9914, 1.0372) | 0.9882 (0.9725, 1.0041) | 1.0022 (0.9913, 1.0132) | 1.0033 (0.9969, 1.0097) |
|  |  |  |  |  |  |  |  |  |
|  | PM_2.5_ | Lag 0 | 1.0007 (0.9973, 1.0041) | 0.9963 (0.9877, 1.0050) | 0.9996 (0.9885, 1.0109) | 0.9955 (0.9887, 1.0023) | 1.0056 (1.0006, 1.0105) | 1.0007 (0.9983, 1.0031) |
|  |  | Lag 1 | 0.9993 (0.9959, 1.0027) | 0.9891 (0.9806, 0.9977) | 0.9927 (0.9815, 1.0040) | 0.9927 (0.9860, 0.9994) | 0.9965 (0.9916, 1.0013) | 0.9968 (0.9944, 0.9992) |
|  |  | Lag 2 | 0.9983 (0.9949, 1.0017) | 0.9913 (0.9831, 0.9997) | 0.9899 (0.9792, 1.0007) | 0.9934 (0.9869, 0.9998) | 0.9980 (0.9934, 1.0027) | 0.9967 (0.9943, 0.9990) |
|  |  | Lag 3 | 0.9974 (0.9940, 1.0008) | 0.9948 (0.9866, 1.0030) | 0.9956 (0.9856, 1.0058) | 1.0018 (0.9955, 1.0081) | 0.9983 (0.9937, 1.0030) | 0.9980 (0.9957, 1.0003) |
|  |  | Lag 0-3 moving average | 0.9984 (0.9941, 1.0026) | 0.9878 (0.9769, 0.9988) | 0.9884 (0.9730, 1.0041) | 0.9906 (0.9807, 1.0006) | 0.9987 (0.9915, 1.0060) | 0.9964 (0.9933, 0.9996) |
|  |  |  |  |  |  |  |  |  |
|  | Ozone | Lag 0 | 1.0051 (0.9956, 1.0146) | 0.9926 (0.9752, 1.0103) | 1.0091 (0.9910, 1.0276) | 0.9965 (0.9814, 1.0117) | 0.9944 (0.9841, 1.0048) | 1.0001 (0.9944, 1.0057) |
|  |  | Lag 1 | 0.9963 (0.9872, 1.0054) | 1.0002 (0.9842, 1.0165) | 1.0085 (0.9915, 1.0258) | 1.0017 (0.9884, 1.0151) | 0.9975 (0.9883, 1.0068) | 0.9990 (0.9938, 1.0042) |
|  |  | Lag 2 | 0.9910 (0.9819, 1.0002) | 0.9852 (0.9690, 1.0018) | 1.0015 (0.9840, 1.0192) | 0.9943 (0.9805, 1.0084) | 0.9998 (0.9903, 1.0095) | 0.9945 (0.9891, 0.9998) |
|  |  | Lag 3 | 0.9963 (0.9873, 1.0054) | 0.9960 (0.9807, 1.0115) | 0.9868 (0.9711, 1.0027) | 1.0001 (0.9875, 1.0127) | 1.0039 (0.9951, 1.0128) | 0.9983 (0.9933, 1.0033) |
|  |  | Lag 0-3 moving average | 0.9944 (0.9818, 1.0071) | 0.9860 (0.9621, 1.0106) | 1.0016 (0.9757, 1.0282) | 0.9949 (0.9709, 1.0195) | 0.9979 (0.9819, 1.0141) | 0.9952 (0.9871, 1.0033) |

**Figure S1** Four-day cumulative associations between ED visits for Alzheimer’s Disease and per IQR increase of PM_2.5_, NO_2_, warm-season ozone in 5 states: California (CA), Missouri (MO), North Carolina (NC), New Jersey (NJ) and New York (NY). Cumulative associations were estimated from a distributed-lag model (lag 0 to lag 3). Pooled estimates were derived from inverse variance weighting. Odds ratios were adjusted by meteorology, holidays, and seasonality; time trends (year, month, day of week) were controlled automatically by the case-crossover design.
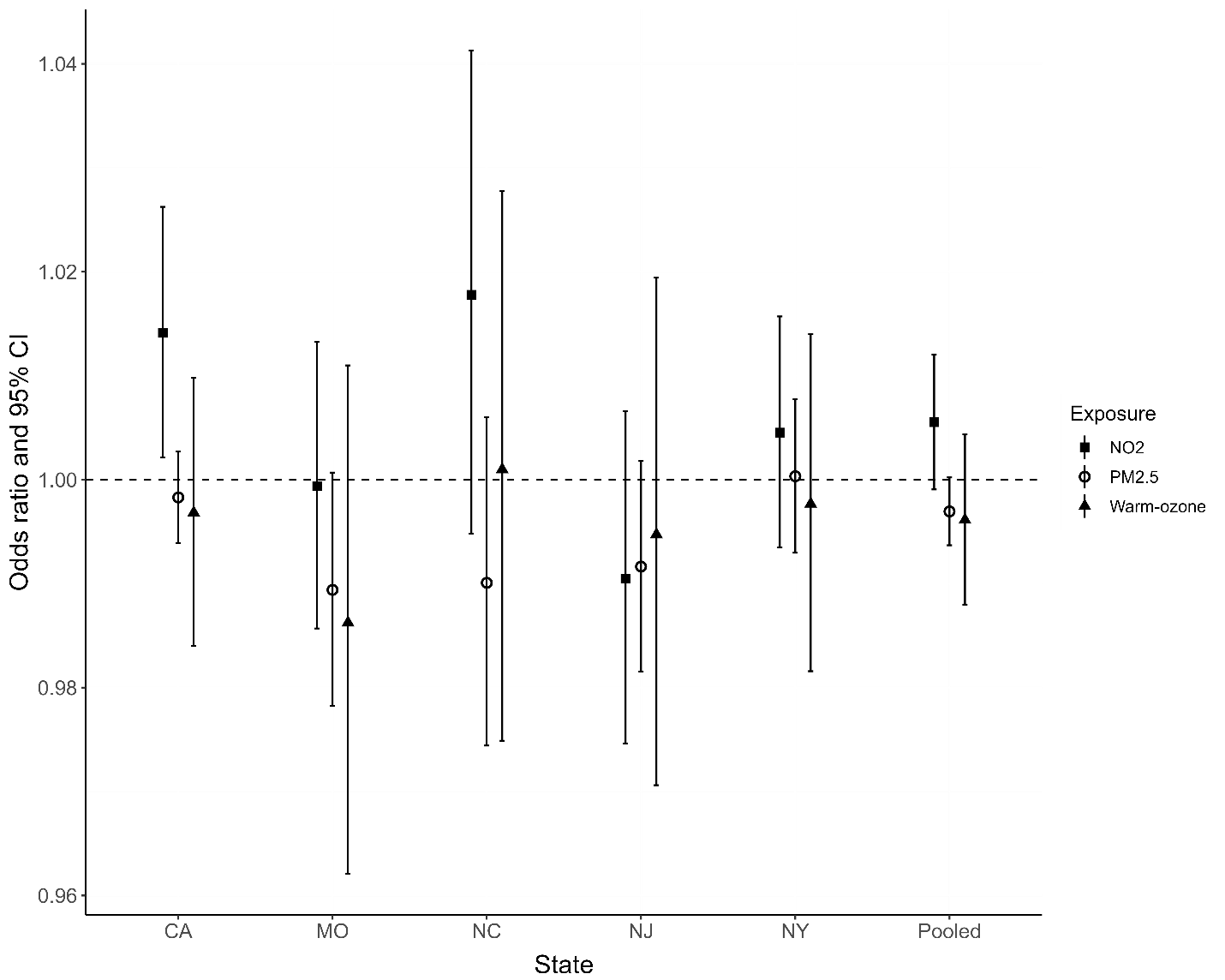


**Figure S2** Lag-specific associations between AD/ADRD ED visits and per IQR increase of PM_2.5_, NO_2_, warm-season ozone (lag 0 to lag 4) in 5 states: California (CA), Missouri (MO), North Carolina (NC), New Jersey (NJ) and New York (NY). All associations were estimated from a distributed-lag model (lag 0 to lag 3). Pooled estimates were derived from inverse variance weighting. Odds ratios were adjusted by meteorology, holidays, and seasonality; time trends (year, month, day of week) were controlled automatically by the case-crossover design.


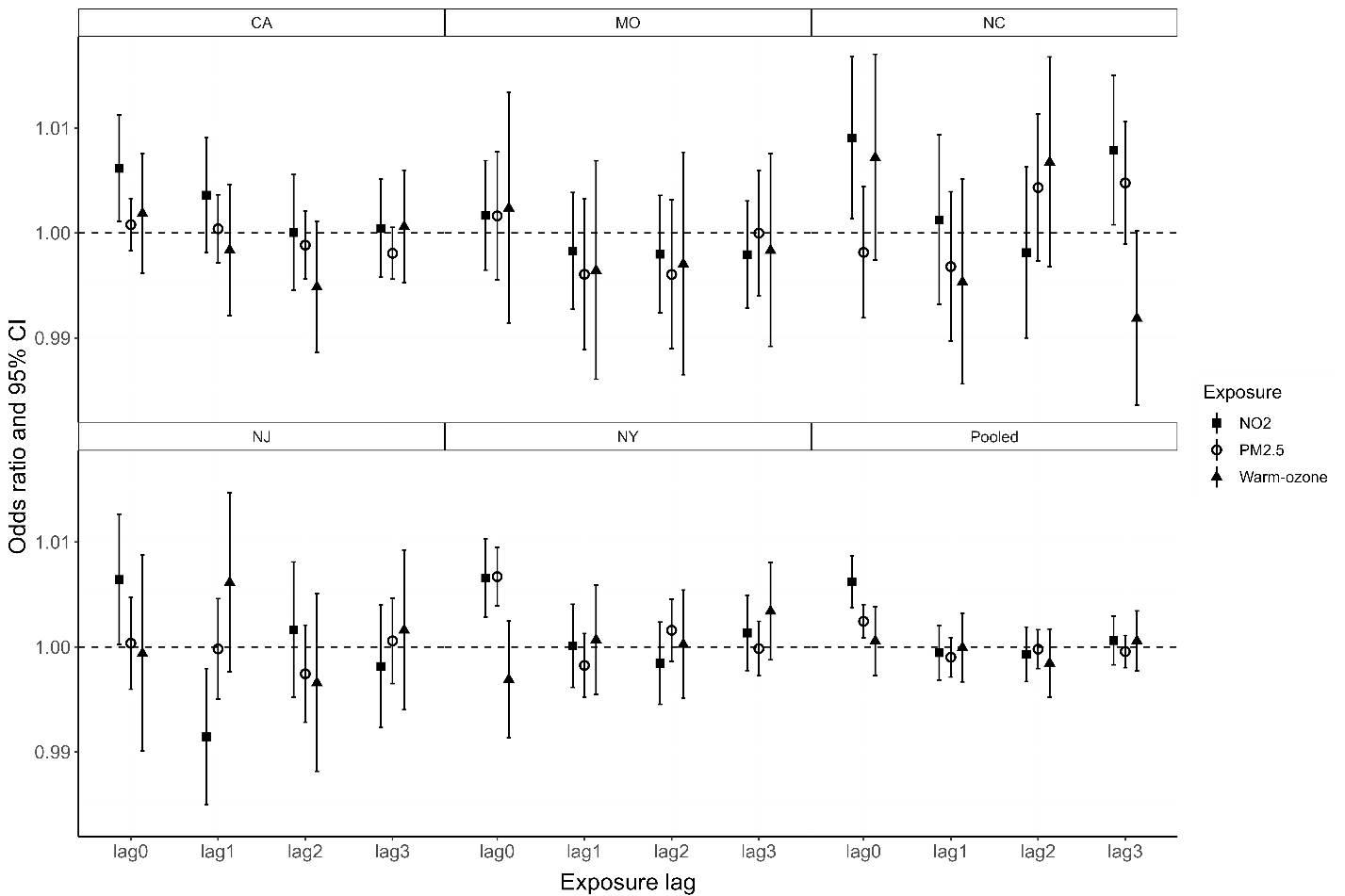


**Figure S3** Short-term associations between AD/ADRD ED visits and per IQR increase in lag0, lag1, lag2, lag3, lag 0-3 moving average PM_2.5_, NO_2_, warm-season ozone in 5 states: California (CA), Missouri (MO), North Carolina (NC), New Jersey (NJ) and New York (NY). Different lagged exposures were fitted separately with conditional logistic regression models. *Lagaver* refers to the lag0 to lag3 day moving average of air pollution levels. Pooled estimates were derived from inverse variance weighting. Odds ratios were adjusted by meteorology, holidays, and seasonality; time trends (year, month, day of week) were controlled automatically by the case-crossover design.
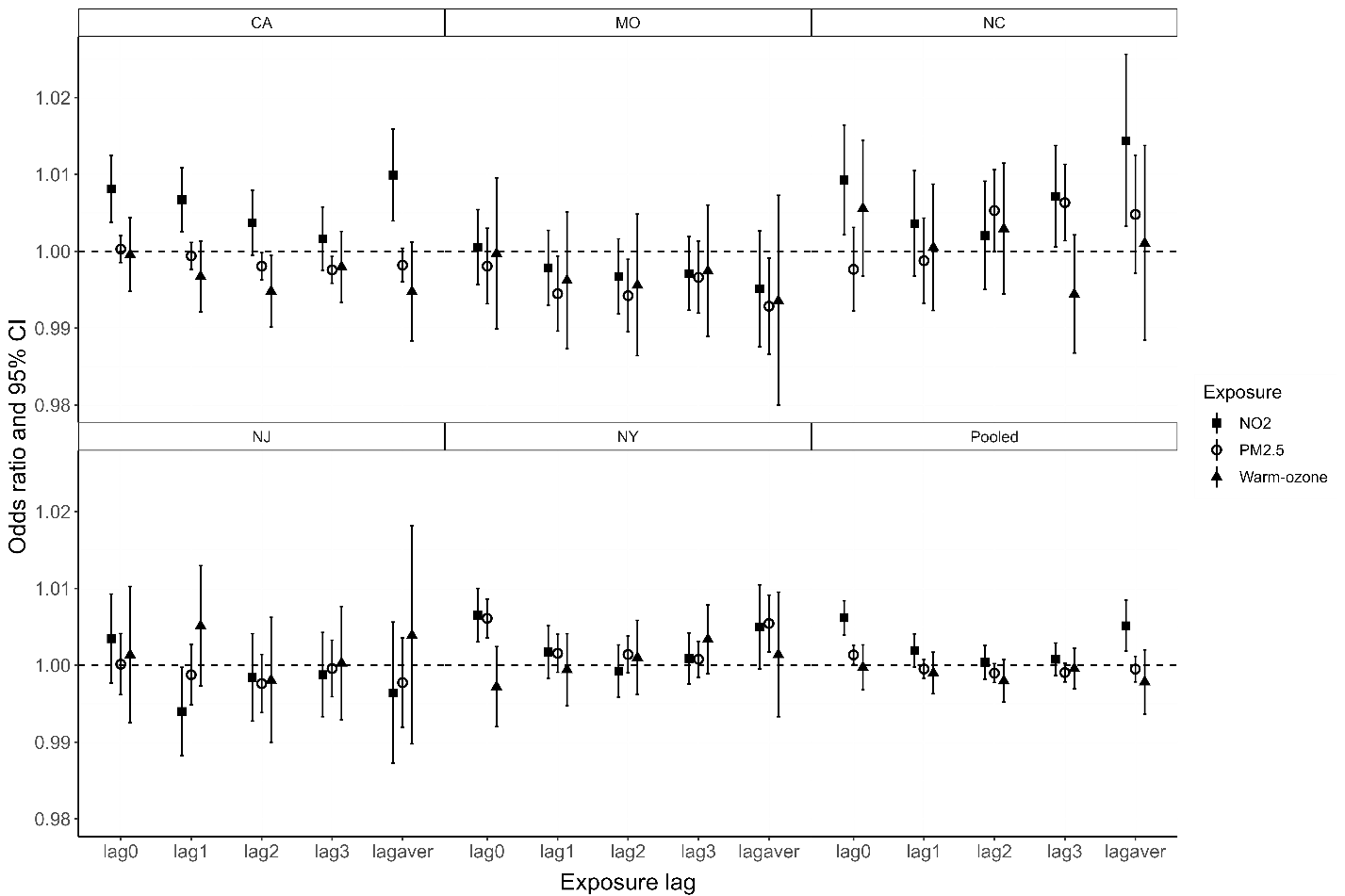


**Figure S4** Comparison of effects of per IQR increase of PM_2.5_, NO_2_, warm-season ozone on AD/ADRD ED visits and non-AD/ADRD ED visits among patients ages 75 years or over in 5 states: California (CA), Missouri (MO), North Carolina (NC), New Jersey (NJ) and New York (NY). Results for non-AD/ADRD ED visits are from cumulative lag models (lag 0 to lag 4 exposures) with conditional Poisson regression, while results for ADRD ED visits are from a cumulative lag models (lag 0 to lag 4 exposures) with conditional logistic regression. Pooled estimates were derived from inverse variance weighting. Odds ratios were adjusted by meteorology, holidays, and seasonality; time trends (year, month, day of week) were controlled automatically by the case-crossover design.
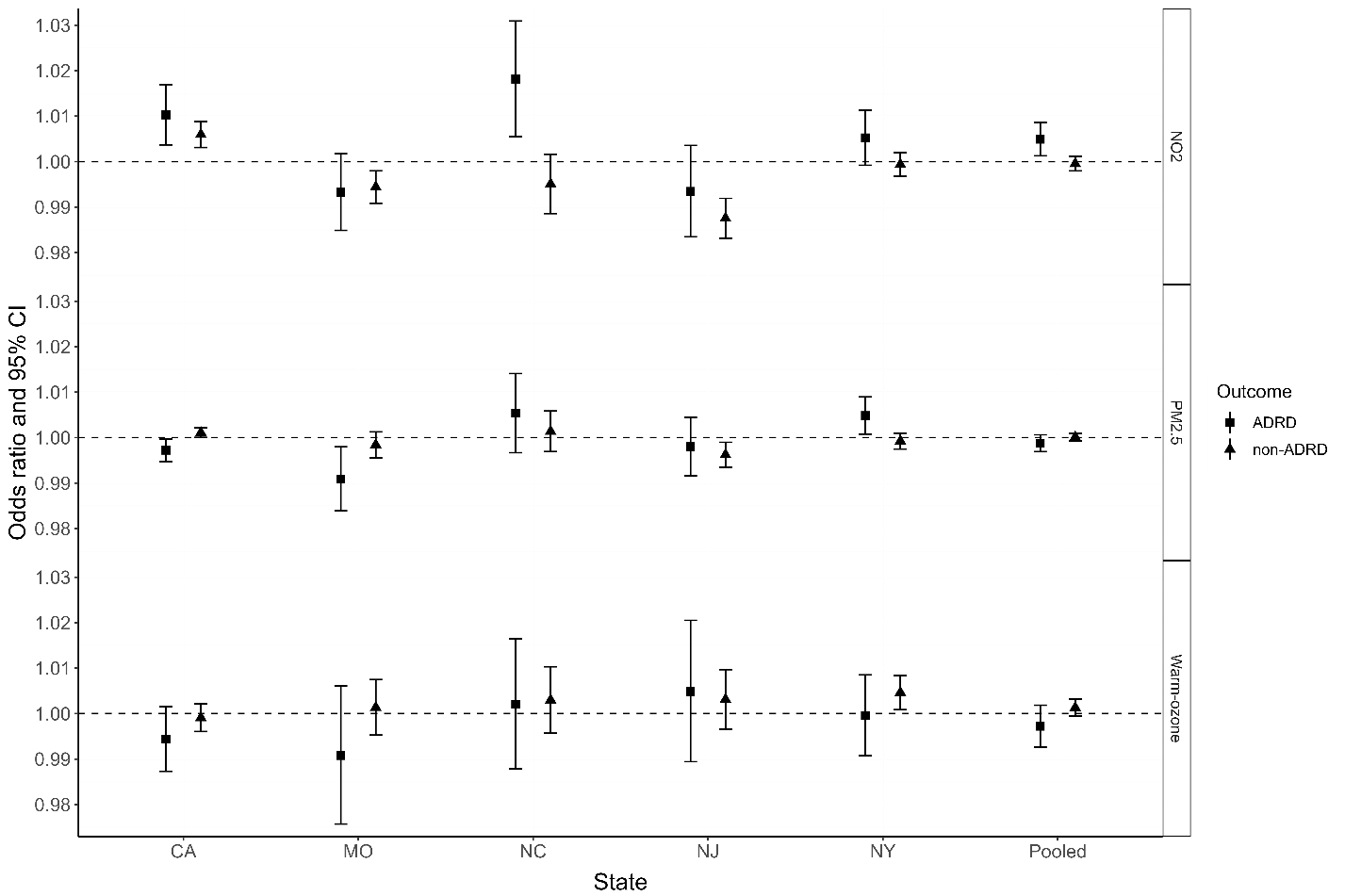

Supplement: Supplementary file 1 [file ee9-7-e237-s001.docx]
